# The Hidden Danger in Endometriosis: Bilateral Pelvic Abscesses Following Fertility Treatment

**DOI:** 10.7759/cureus.86707

**Published:** 2025-06-25

**Authors:** Diya E Viju, Diana George, Sandesh Kade, Vipin Dagar, Robin M Kuruvilla, Ranjita Das

**Affiliations:** 1 Radiology, Burjeel Medical City, Abu Dhabi, ARE; 2 Obstetrics and Gynaecology, Burjeel Medical City, Abu Dhabi, ARE; 3 Radiology, Burjeel Hospital, Abu Dhabi, ARE; 4 Radiology, LLH Hospital, Abu Dhabi, ARE

**Keywords:** assisted reproductive technology, endometriosis, in vitro fertilization, pelvic inflammatory disease, pelvic mri, tubo-ovarian abscess

## Abstract

This case report underscores the crucial role of imaging and radiology in the timely diagnosis of a rare but serious complication associated with assisted reproductive technology (ART). A 34-year-old woman with a long-standing history of endometriosis and secondary infertility developed bilateral tubo-ovarian abscesses (TOAs) following in vitro fertilization (IVF). Her presentation included nonspecific gastrointestinal and pelvic symptoms that delayed diagnosis. However, a multimodal imaging approach, combining transvaginal ultrasound, contrast-enhanced computed tomography (CT), and pelvic magnetic resonance imaging (MRI), proved critical in identifying a superimposed infection on chronic adnexal disease. Subsequent surgical exploration confirmed the diagnosis of TOA and hematopyosalpinx. This case illustrates the diagnostic complexity of differentiating infectious processes from endometriotic flare-ups in the post-ART setting and highlights the indispensable role of early and advanced imaging in guiding clinical management, preventing sepsis, and preserving fertility.

## Introduction

Endometriosis is a chronic, estrogen-dependent inflammatory condition that affects approximately 10% of women of reproductive age. It is a major contributor to pelvic pain, dysmenorrhea, dyspareunia, and infertility. In advanced cases, endometriosis can lead to the formation of endometriomas, hydrosalpinx, chronic pelvic adhesions, and even bowel or urinary tract involvement. These anatomical distortions significantly complicate fertility treatment and surgical interventions [1].

In women undergoing assisted reproductive technology (ART), such as in vitro fertilization (IVF), pre-existing endometriosis and tubal disease increase the risk of superimposed infection, including tubo-ovarian abscesses (TOAs), particularly after intrauterine instrumentation or hormone stimulation. These infections, if not identified early, can result in sepsis, infertility, or the need for radical surgical intervention [2].

This case describes a 34-year-old woman with long-standing endometriosis and secondary infertility who developed bilateral TOAs following ART. She presented with recurrent pelvic pain, gastrointestinal symptoms, and systemic signs of infection. Her clinical journey underscores the importance of a multimodal imaging approach, including ultrasound, contrast-enhanced CT, and pelvic MRI, in not only detecting complex adnexal pathology but also in differentiating between gynecologic and gastrointestinal causes of pelvic pain.

The case is significant not only because of the rare evolution of pelvic endometriosis into abscess formation post-IVF but also because it underscores the importance of early imaging, multidisciplinary care, and prompt surgical management in preserving fertility and preventing life-threatening complications [3].

## Case presentation

A 34-year-old woman of South Asian descent with a history of dysmenorrhea and endometriosis presented with persistent pelvic pain and secondary infertility. She was initially found to have an endometrial polyp, for which she was placed on oral contraceptive pills for medical management. During the same period, she experienced worsening symptoms and was diagnosed with chronic salpingitis and a complex adnexal cyst, characterized as likely hemato- or hydrosalpinx, on pelvic ultrasound.

Subsequently, she initiated fertility planning and underwent further evaluation. An MRI of the pelvis was performed, revealing a 5.0 x 2.2 cm complex cyst in the left ovary. The lesion demonstrated T1 hyperintensity, indicating hemorrhagic content, and T2 shading, both characteristic of an endometrioma. The cyst lacked septations or solid components. Additional findings included bilateral hydrosalpinx, with the left fallopian tube distended and containing T1 hyperintense and T2 heterogeneous content, consistent with proteinaceous or hemorrhagic debris. A focal 8 mm polypoidal lesion was visualized within the endometrial canal.

Following this, she began ART therapy at a fertility clinic. As part of the preparatory steps, she underwent hysteroscopy with polypectomy, endometrial sampling, and dilatation and curettage. After embryo transfer, she unfortunately experienced a missed abortion, which was managed medically with mifepristone. Eight months following this event, she developed lower abdominal pain, diarrhea, fever, and vomiting while still on hormonal support for her infertility.

An abdominal ultrasound at this point revealed thickening of the distal colon (up to 4.5 mm) and a 6 x 5 cm cystic lesion in the left ovary. The cyst appeared thin-walled and fluid-filled, raising suspicion for either a persistent endometrioma or superimposed infectious etiology. Empirical antibiotics were initiated. Inflammatory markers were notably raised, with a CRP of 298 mg/L, indicating a possible pelvic or intra-abdominal inflammatory process.

The patient's symptoms persisted, and she presented at the emergency department with clinical signs suggestive of acute appendicitis. A contrast-enhanced CT scan was done, which showed a thick-walled, complex cystic mass in the left adnexal region with peripheral enhancement. The left fallopian tube appeared dilated, fluid-filled, and surrounded by fat stranding. The appendix measured 7-8 mm in diameter, with mild mural thickening and contrast enhancement, but lacked definitive signs of perforation or appendicolith, making it less likely to be the primary cause.

She was managed conservatively for acute pelvic inflammatory disease (PID), presumed secondary to ascending infection complicating pre-existing hydrosalpinx and endometriosis. However, with an ongoing fever, elevated CRP (259 mg/L), and increased WBCs, she was referred to gynecology for further evaluation.

At the gynecology clinic, she reported worsening dysmenorrhea, dyspareunia, bloating, and bowel irregularities, particularly alternating constipation and diarrhea accompanied by rectal pain. The laboratory workup results were significant, as shown in Table 1.

**Table 1:** Abnormal laboratory values of the patient indicating active inflammation. CRP: C-reactive protein; WBC: white blood cell count; Hb: hemoglobin; CA-125: cancer antigen 125; TOA: tubo-ovarian abscess; PT: prothrombin time; APTT: activated partial thromboplastin time

Laboratory Parameter	Result	Reference Range	Interpretation
C-Reactive Protein (CRP)	205 mg/L	<10.0 mg/L	Elevated; suggestive of active inflammation/infection
Amylase	2000 U/L	30–110 U/L	Markedly elevated; consider pancreatitis or peritonitis
White Blood Cells (WBC)	20.76 × 10⁹/L	4.5–11.0 × 10⁹/L	Elevated; indicative of systemic inflammatory response
Hemoglobin (Hb)	9.8 g/dL	12–16 g/dL	Low; indicative of mild to moderate anemia
CA-125	47 U/mL	0–35 U/mL	Mildly elevated; seen in endometriosis, TOA, malignancy
Prothrombin Time (PT)	Prolonged	-	Suggestive of coagulation abnormality
Activated Partial Thromboplastin Time (APTT)	Prolonged	-	Indicates an intrinsic pathway abnormality or inflammation
Urine Microscopy	Microscopic hematuria	-	Presence of red blood cells in urine, suggestive of urinary tract involvement

A transvaginal sonography revealed a mid-positioned, bulky uterus with a distorted endometrial stripe and an obliterated pouch of Douglas, which could indicate dense pelvic adhesions. A 7 x 3 cm left ovarian cyst with mixed echogenicity was seen, with absent Doppler flow, raising concern for infectious or ischemic complications.

**Figure 1:**
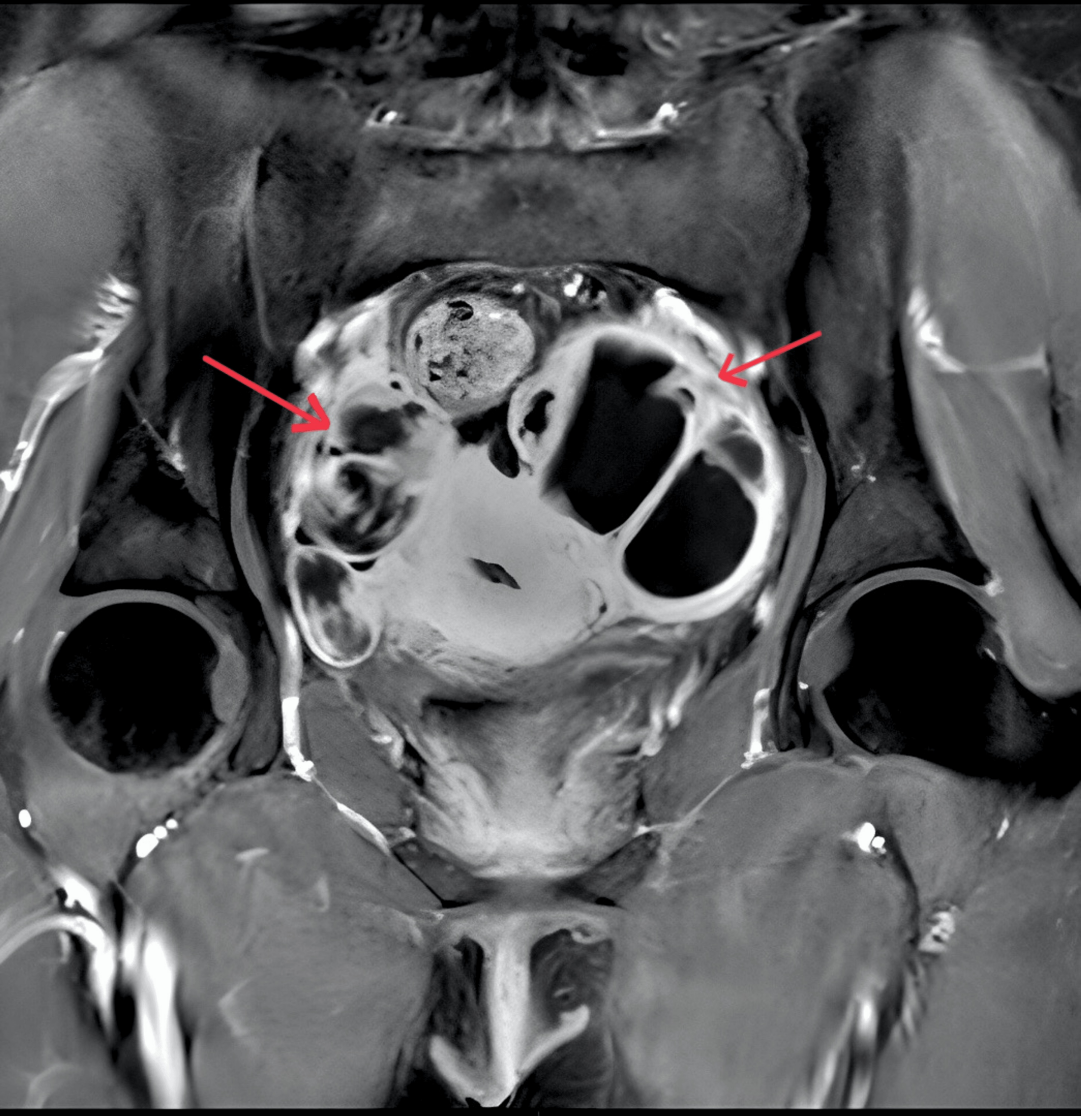
A contrast-enhanced coronal MRI shows a peripherally enhancing, multiloculated collection in the bilateral adnexa, consistent with tubo-ovarian abscesses (arrows).

**Figure 2:**
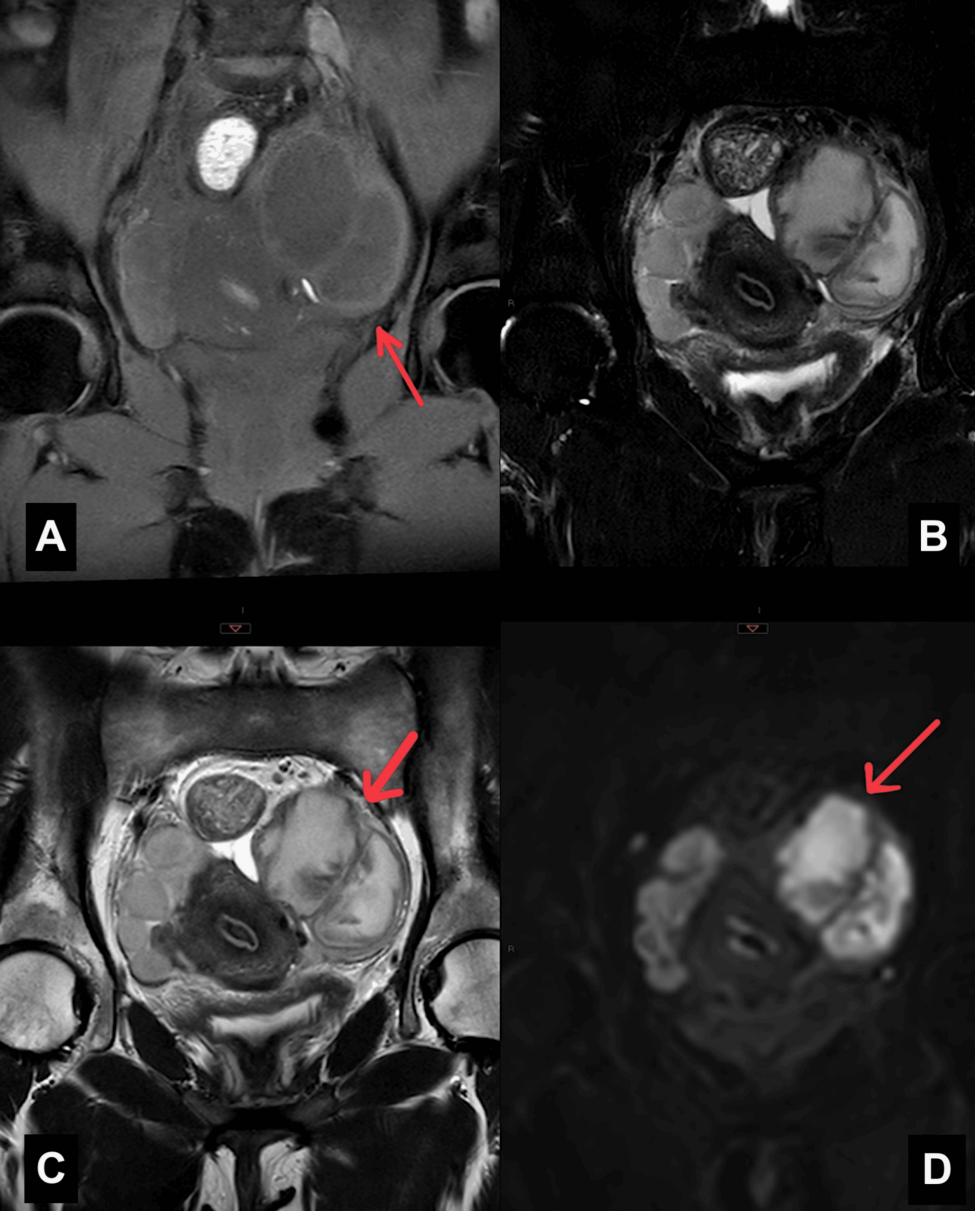
(A) T1, (B) T1 fat-saturated, (C) T2, and (D) DWI images of the pelvis showing a hypointense signal on T1, an intermediate signal on T2, and restricted diffusion on DWI, suggestive of a TOA. T1: T1-weighted MRI image; T1 fat-saturated: T1 image with fat signal suppressed to highlight lesions; T2: T2-weighted MRI image; DWI: diffusion-weighted imaging; TOA: tubo-ovarian abscess

Prior to the scheduled follow-up imaging, the patient re-presented to the emergency department with progressively worsening lower abdominal pain, rebound tenderness, and clinical signs indicative of peritonism. A pelvic MRI with and without contrast was performed, revealing several significant findings. Bilateral pelvic abscesses were identified, characterized by multiloculated fluid collections (Figure 1). Additionally, both adnexal regions exhibited T2 shading and T1 hyperintensity, findings suggestive of chronic hemorrhagic content complicated by superimposed infection (Figure 2). There was also peritoneal reactive free fluid present, consistent with localized peritonitis. On the right side, multiple complex cystic lesions were observed within the ovary, with the largest measuring 3.5 x 2.3 cm. This lesion demonstrated central T1 hyperintensity and a peripheral hypointense rim on T2-weighted imaging, consistent with an organized hematopyosalpinx (Figure 2).

**Figure 3:**
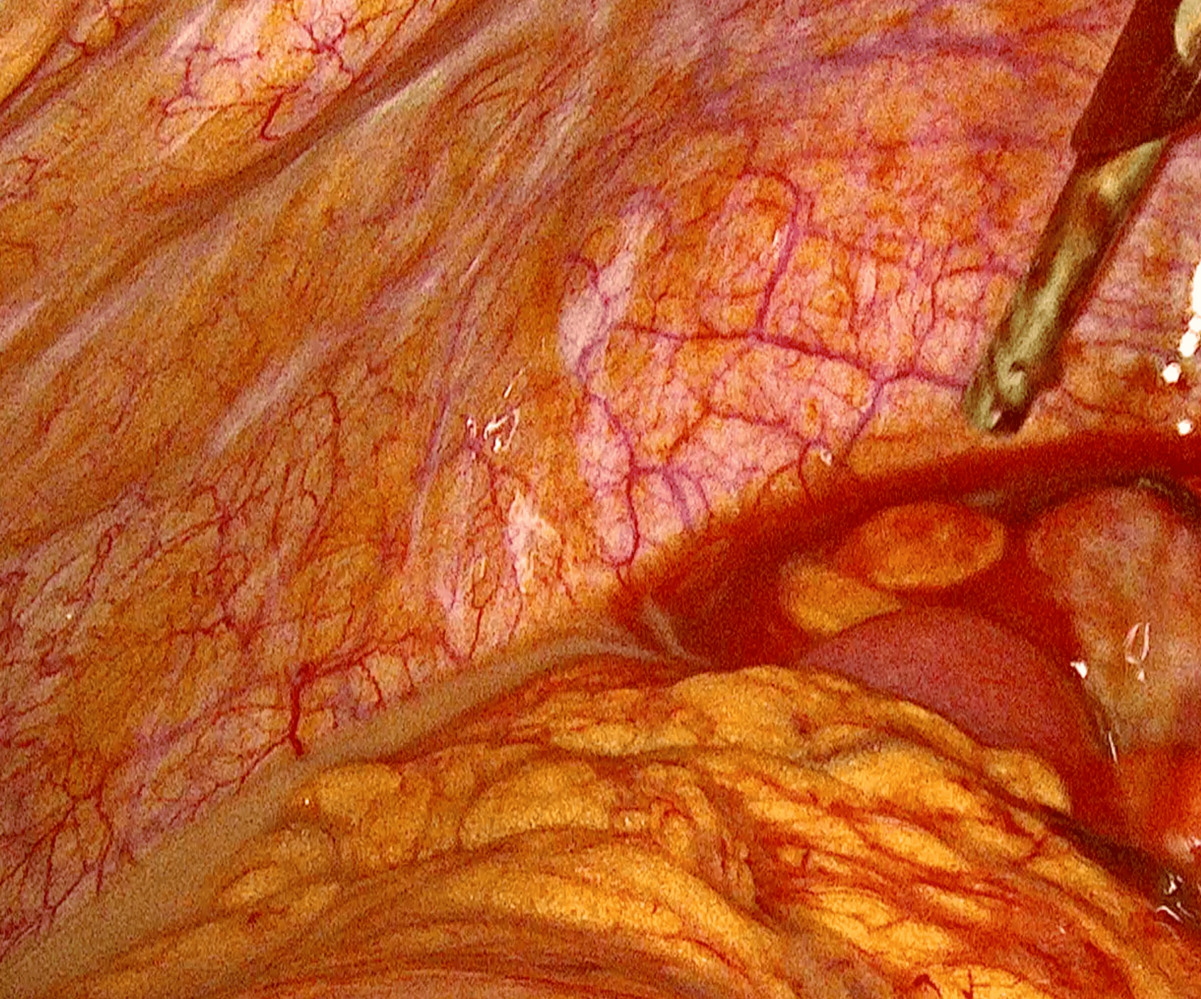
Laparoscopic view showing purulent and hemorrhagic collections from a right fallopian tube abscess, consistent with a tubo-ovarian infection.

**Figure 4:**
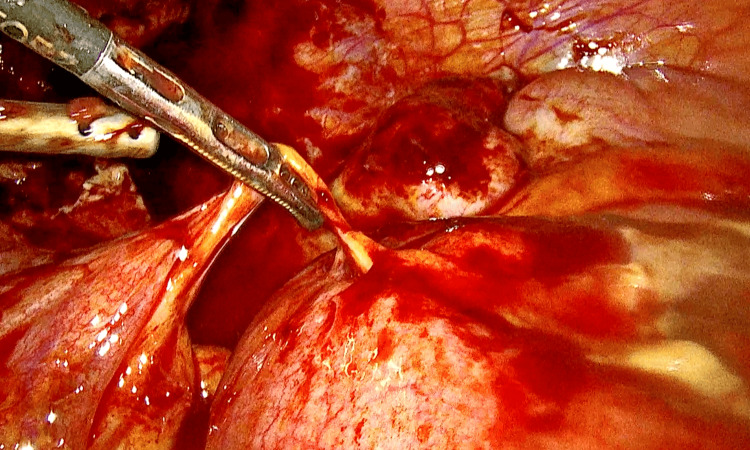
The laparoscopic view reveals dense fibrous adhesions within the abdominal cavity.

The patient underwent emergency diagnostic laparoscopy with extensive adhesiolysis and enterolysis. The patient was found to have multiple dense intra-abdominal adhesions, necessitating meticulous dissection to restore normal anatomy (Figure 3). Intraoperatively, the omentum appeared thickened and "cakey," densely adherent to the anterior abdominal wall, right adnexa, and the pouch of Douglas. A left TOA, with purulent collection engulfing the ovary and fallopian tube, was identified and drained. On the right side, a hematopyosalpinx was confirmed and decompressed (Figure 4). The appendix appeared mildly edematous but was preserved, as it was not identified as the primary source of infection.

**Figure 5:**
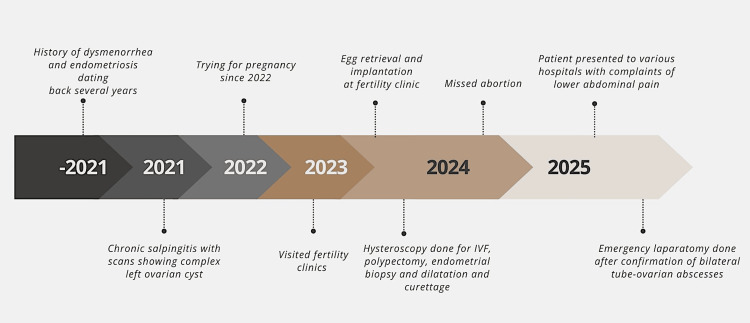
Chronological timeline summarizing the patient's gynecologic and reproductive history, clinical presentations, and interventions leading up to the definitive surgical management. Image created by the authors.

The patient tolerated surgery well. Postoperatively, she showed rapid clinical improvement, with resolution of pain and gastrointestinal symptoms. CRP levels decreased significantly to 2.3 mg/L, and WBC counts normalized. She was discharged in stable condition with a tailored plan for continued gynecologic care, suppression of endometriosis, and fertility counseling, given her reproductive history and the structural compromise of both adnexae. For clarity and continuity, a clinical timeline has been constructed to delineate the sequence of events, diagnostic evaluations, and therapeutic interventions leading up to the surgical procedure (Figure 5).

## Discussion

This case highlights the complex clinical challenges in managing patients with advanced endometriosis undergoing IVF, particularly the risk of developing serious infectious complications such as PID and TOA.

Endometriosis is a chronic, inflammatory gynecological condition that not only causes debilitating pelvic pain but also plays a major role in causing infertility [1]. It alters pelvic anatomy, impairs tubal function, affects oocyte quality, and creates a hostile peritoneal environment, all of which compromise natural conception [4]. As a result, many women with moderate to severe endometriosis eventually turn to ART to conceive. ART includes various fertility treatments that manage both eggs and sperm, with IVF being the most common. IVF involves hormonal stimulation of the ovaries, retrieval of multiple oocytes, IVF with sperm, and subsequent transfer of selected embryos into the uterus [5]. Endometriosis affects 10%-15% of all reproductive-age females and 70% of women with persistent pelvic pain [6].

However, the pathophysiological changes brought about by endometriosis, such as endometriomas, hydrosalpinx, and chronic pelvic adhesions, pose unique challenges during ART [7]. In women with pre-existing tubo-ovarian disease, embryo transfer procedures, endometrial manipulation, or hormone-induced changes in pelvic vascularity may predispose them to ascending infections, culminating in severe complications such as TOA, peritonitis, or even sepsis. These infections are rare but potentially life-threatening, especially when diagnosis is delayed due to overlapping symptoms with endometriosis flares or gastrointestinal disturbances [8].

The chronic nature of endometriosis often necessitates long-term management strategies beyond acute infection control. Medical therapies such as GnRH (gonadotropin-releasing hormone) agonists, progestins, or combined oral contraceptives can suppress disease activity but are typically contraindicated during fertility treatment [9,10]. Surgical excision of endometriotic lesions and cystectomy for endometriomas may improve pain and fertility but carry a risk of reduced ovarian reserve [11].

In this case, the patient underwent ART in the setting of chronic endometriosis, bilateral hydrosalpinx, and prior adnexal disease. She subsequently developed bilateral TOAs, a rare but serious complication, presenting with nonspecific symptoms such as abdominal pain, fever, vomiting, and altered bowel habits. The clinical ambiguity necessitated a high index of suspicion and a multimodal imaging approach to arrive at the correct diagnosis.

Diagnosing PID or TOA in a woman with endometriosis post-IVF can be particularly challenging due to overlapping symptoms such as pelvic pain, bloating, and gastrointestinal problems. Here, advanced cross-sectional imaging played a pivotal role in establishing the diagnosis and guiding management. Ultrasound was the initial modality, identifying complex ovarian cysts and free fluid, but lacked the specificity in distinguishing between hemorrhagic endometriomas and abscesses [12]. CT imaging offered a broader abdominal context and ruled out appendicitis or bowel perforation.

The turning point in diagnosis came with an MRI of the pelvis, which provided excellent soft-tissue contrast and anatomical delineation. The presence of T1 hyperintensity and T2 shading in the adnexal masses confirmed the presence of hemorrhagic content, characteristic of endometriomas, while adjacent multiloculated, thick-walled fluid collections with peripheral enhancement strongly suggested superimposed infection [12]. These findings prompted emergency diagnostic laparoscopy, which revealed extensive adhesions, a pus-filled left TOA, and a right-sided hematopyosalpinx, confirming the imaging findings and guiding surgical intervention.

This case illustrates the life-saving role of timely imaging in women with complex gynecologic conditions undergoing ART. Inflammatory markers and clinical symptoms may be nonspecific, especially in patients already experiencing chronic pelvic pain from endometriosis. Delays in imaging and diagnosis can lead to rupture of abscesses, systemic sepsis, and loss of ovarian function, further compromising fertility and risking maternal health. Therefore, clinicians must maintain a high index of suspicion for TOA or pelvic sepsis in endometriosis patients presenting with acute symptoms post-IVF. Table 2 provides a comparative overview of the benefits and limitations of imaging versus inflammatory markers, highlighting the essential roles of both in the accurate and timely diagnosis of pelvic inflammatory disease [13].

**Table 2:** A comparison between imaging studies and inflammatory markers in the diagnosis of PID and TOAs. CT: computed tomography; MRI: magnetic resonance imaging; TOA: tubo-ovarian abscess; PID: pelvic inflammatory disease

Aspect	Imaging Studies	Inflammatory Markers
Role	Definitive diagnosis and assessment of abscess size, location, and complications.	Indicate the presence and severity of infection; monitor treatment response.
Sensitivity	High; especially with advanced imaging techniques like MRI and CT.	Moderate; varies with the marker and its threshold.
Specificity	High; specific findings associated with TOA.	Low; can be elevated in various infectious and inflammatory conditions.
Utility	Essential for confirming diagnosis and guiding treatment decisions.	Useful for initial assessment and monitoring; not definitive for diagnosis.
Limitations	May require contrast agents; availability may vary; potential radiation exposure with CT.	Cannot localize or visualize abscess; nonspecific elevation in various conditions.

## Conclusions

This case illustrates the complex interplay between endometriosis, ART, and pelvic infection, and how this triad can lead to life-threatening complications if not promptly recognized and managed. It reinforces the critical importance of infection risk stratification prior to ART, vigilant post-procedural monitoring, and the early use of advanced imaging to ensure diagnostic clarity.

Radiological imaging was crucial for identifying the evolving pathology and guiding timely surgical decisions, preventing sepsis and preserving fertility. While the initial ultrasound suggested a benign endometriotic cyst, follow-up CT and MRI revealed superimposed pelvic infection, bilateral TOAs, and peritoneal inflammation. In women with endometriosis undergoing assisted reproduction, a high index of suspicion for complications like TOAs is essential. This case also highlights the value of multidisciplinary collaboration among gynecology, radiology, infectious disease, and fertility specialists in managing complex gynecologic and reproductive conditions.
